# 

**Published:** 1988-08

**Authors:** 


W1 BR323

V.103 NO.3 1988

C.D1---------SEQ: B33740000

TI: BRISTOL MEDICO-CHIRURGICAL

JOURNAL

### BRISTOL MEDICO-CHIRURGICAL JOURNAL

VOLUME 103(iii) AUGUST 1988

Domestic Medicines
The trainee in the General practice Surgery.
Sweet's syndrome
Aneurysm of the interatrial septum.
The Southmead Lithotripter.
Surgical Safari. etc. etc.

NATIONAL LIBRARY
JAN 31 1989
OF MEDICINE

THE MEDICAL JOURNAL OF THE SOUTH WEST

